# Attention for and awareness of anemia in adolescents in Karnataka, India: A qualitative study

**DOI:** 10.1371/journal.pone.0283631

**Published:** 2023-04-05

**Authors:** Bronwen Gillespie, Geetanjali Katageri, Shumona Salam, Umesh Ramadurg, Shrinivas Patil, Jayaraj Mhetri, Umesh Charantimath, Shivaprasad Goudar, Ashwini Dandappanavar, Chandrashekhar Karadiguddi, Ashalata Mallapur, Phaniraj Vastrad, Subarna Roy, Basavaraj Peerapur, Dilly Anumba

**Affiliations:** 1 Faculty of Medicine, Dentistry and Health, The University of Sheffield, Sheffield, South Yorkshire, United Kingdom; 2 Department of OBG, S N Medical College and HSK Hospital and Research Centre, Bagalkot, Karnataka, India; 3 Department of Community Medicine, S N Medical College and HSK Hospital and Research Centre, Bagalkot, Karnataka, India; 4 Department of Community Medicine, KLE Academy of Higher Education and Research J N Medical College, Belagavi, Karnataka, India; 5 KLE Academy of Higher Education and Research J N Medical College, Belagavi, Karnataka, India; 6 Women’s and Children’s Health Research Unit, KLE Academy of Higher Education and Research, Belagavi, Karnataka, India; 7 Model Rural Health Research Unit, Sirwar, Raichur, Karnataka, India; 8 ICMR-NITM, Belagavi, Karnataka, India; 9 Raichur Institute of Medical Sciences, Raichur, Karnataka, India; Pokhara University, NEPAL

## Abstract

Between 1990 and 2016 the number of adolescents with anemia world-wide increased by 20% to almost one in four. Iron deficiency in adolescence results in compromised growth, decreased cognitive function, and depressed immune function, and can increase the risk of negative outcomes in pregnancy, especially in the case of young adolescents. In India, despite several decades of governmental investment in anemia prevention and treatment, more than half of women of reproductive age are anemic, with rates even higher in the adolescent population. Although awareness of adolescence as a nutrition-sensitive developmental stage is increasing, there is a lack of qualitative research on the perspectives of adolescents and families on anemia and related services. In this study, we explored the issues influencing adolescents’ awareness of anemia in three rural areas of Karnataka. Sixty-four in-depth interviews and six focus group discussions were conducted with adolescents (those who had never been pregnant, pregnant adolescents, and young mothers), community members, and nutrition-related service providers in the health and education sectors. An inductive analytical approach was used. We found that adolescent girls, particularly those who have not experienced pregnancy or motherhood, had very low awareness of anemia. State programs including school-based distribution of iron and folic acid supplements and nutrition talks were not seen to be resulting in knowledge and acceptance of the importance of preventing anemia. Pregnancy represents a turning point in which adolescents are systematically tested for anemia as part of routine antenatal care, increasing their awareness of, and access to, treatment for the condition. At the same time, pregnancy represents to family and community a period to insist on a nutritious diet. For progress in anemia reduction to be made, improved age-appropriate measures specific for adolescence are required. Improving school-based nutrition outreach is an important opportunity to reach adolescents.

## Introduction

Anemia is associated with increased morbidity and mortality, poor birth outcomes, and impaired cognitive development [1]. It is a widespread global health challenge: in 2019, anemia prevalence was 29.9% in women of reproductive age, equivalent to over half a billion women aged 15–49 years [2]. Iron deficiency is the most common cause of anemia, estimated to contribute to approximately 50% of anemia cases globally [1]. This is especially important for female adolescents, as iron requirements increase due to rapid growth and the onset of menstruation [3–5]. From 1990 to 2016 the absolute number of adolescents with anemia world-wide increased by 20% to almost one in four [6]. Iron deficiency in adolescence results in compromised growth, decreased cognitive function, and depressed immune function [7]. Anemia in pregnancy, especially in the case of young adolescents, can increase the risk of adverse outcomes [7]. Considering the existence of these nutrition risks for female adolescents, there is a dearth of adolescent-specific nutrition programming [8,9]. Despite widespread knowledge of the importance of iron-deficiency anemia (IDA) in health policy circles, and the existence of evidence-based recommendations regarding iron supplementation for those at risk [3], preventing it has proven to be challenging [10], especially in low-and-middle-income countries (LMICs) [11,12].

In India, despite several decades of government investment in anemia prevention and treatment, high rates continue. More than half of women of reproductive age are anemic, according to the 2019 National Family Health Survey 5 [13] which shows that rates of anemia in adolescents aged 15–19 years have increased to 60.2%, compared to the 54.1% reported in the NHFS 4 data in 2016 [14]. Research in India shows that socioeconomic disadvantage leaves people at risk of IDA [11,15] due to lack of access to a diverse diet [16], poor environmental sanitation and unsafe drinking water [17]. Vulnerable groups, such as pregnant women, children, and the poor, are more likely to face other risk factors such as repeated hookworm infestation and multiple micronutrient deficiencies [15]. Studies point to the challenges facing adolescent-oriented anemia prevention programs—ensuring access, coverage and sustainability [4,15,17,18]. Furthermore, research in LMIC contexts as a whole [4,9,19–21] suggests that adolescent and community lack of awareness of anemia and of their own nutrition status is one of the factors limiting progress in anemia prevention. Some Indian studies have observed low IDA awareness in adolescents [21] low adolescent IFA consumption and low community awareness regarding anemia, especially in young people and families in vulnerable urban communities [20]. However, there is little research on anemia awareness and experience with anemia prevention services from the perspective of adolescent girls themselves and those associated closely with them. This research aims to address this gap so to contribute to improving the orientation of nutrition services specifically for adolescent girls.

This study aimed to comprehend adolescents’ awareness of anemia, their access to and use of nutrition services and information, and the perspectives of community members and service providers, in three rural areas of Karnataka, India.

## Methods

This qualitative study (from emic and etic perspectives) was carried out in rural Bagalkot, Belagavi and Raichur districts in north Karnataka, between November 2020 and March 2021. Researchers aimed to select rural settings located at least 20 km. from the district headquarters. The three districts chosen are geographically adjacent, with similar customs and diets, and comparable health outreach programs. Local level government health personnel helped identify potential participants. Ten researchers (7 men, 3 women) carried out semi-structured qualitative interviews with female adolescents aged 13 to 19, health care providers, program managers and school teachers, for a total of 64 in-depth interviews (IDI). Female adolescents included new mothers and pregnant adolescents, who will be referred to as “adolescent mothers”, and adolescents who were not and had never been pregnant, who will be referred to as “adolescents”. The study focused on adolescent females rather than males, taking into account the higher rates of anemia in adolescent girls. Most adolescents were interviewed by female researchers to facilitate their comfort with the process. Six focus group discussions (FGD) were also held with family members and community leaders. Details on data collection, including the range of roles and numbers of service providers, are outlined in Table 1. Table 1 also provides some basic information about community members who participated in the FGD in each location. The family members included fathers, mothers, mothers-in-law, and ranged in age from 28 to 52 years. The community leaders’ ages ranged from 31 to 80 years.

**Table 1:** Data collection.

	Raichur	Bagalkot	Belagavi	Total
**IDIs**				
**Non-pregnant adolescents**	**8**	**6**	**6**	**20**
**Adolescent mothers**	**7**	**6**	**6**	**19**
**Health care providers**	**6**	**5**	**5**	**16**
Medical officers (MO, Ayush MO)	2	1	1	
Auxiliary nurse midwives* (ANM)	1	1	1	
Accredited Social health activists (ASHA)	1	1	1	
Anganwadi worker (AWW)	1	1	1	
Male health workers (MHW)	1	1	1	
**Programme managers**	**2**	**2**	**2**	**6**
Reproductive and child health Officers (RCHO)/ District Health Officer (DHO)	1	1	1	3
Child development project managers (CDPO)	1	1	1	3
**School teachers**	**1**	**1**	**1**	**3**
**Total IDIs**				**64**
**FGDs**				
Community leaders	1	1	1	3
Family members (adults with adolescent daughters)	1	1	1	3
**Total FDGs**				**6**
Female participants	8	6	5	19
Male participants	4	6	7	17
**Total participants in FDGs**				**36**

*now called Primary Health Care Officers.

Interview guides organized by thematic topics were prepared for adolescents, pregnant adolescents and adolescent mothers and community members focusing on food habits, nutrition awareness, and perceptions of related government schemes. Interviews for service providers explored their views on nutrition-related programs for adolescents, and perceptions of challenges. Before initiating each interview, informed consent was obtained from participants, and in the case of minors (under 18 years of age), from the parent/guardian as well. The minors were told clearly that they need not participate in the interview if they did not wish to, even if their parent/ guardian had consented. Informed consent forms were in Kannada, the local language. For non-literate participants, the form was read out. Interviews were conducted in Kannada, audio recorded, and transcribed and translated to English by the team.

The research protocol was approved by the institutional ethics committees at the three study sites (Institutional Ethics Committee, KLE Academy of Higher Education and Research, Belagavi, Raichur Institute of Medical Sciences Institutional Ethics Committee and SNMC Institutional Ethics Committee on Human Subjects Research, S Nijalingappa Medical College and HSK Hospital and Research Centre, Bagalkot) and also at the University of Sheffield. The Health Ministry’s Screening Committee at the Indian Council for Medical Research approved the project. The trial was registered with the Clinical Trial Registry of India (CTRI/2020/09/027515) permission was also granted by the Karnataka State Ethics Committee to enable the participation of the health centres and staff. In regards to inclusivity in global research, additional information regarding the ethical, cultural, and scientific considerations specific to inclusivity in global research is included in the Supporting Information.

Data was analyzed using an inductive thematic approach. Transcripts were read by team members to develop an initial coding framework which was updated after the first few interviews were entered, using NVivo Software. Interviews with adolescents and FDGs with community members were coded separately from service providers, based on the interview guides. Coded sections were reviewed to generate key-words and themes, and team meetings were held to cross-check findings.

## Results

Firstly, we describe the participants in the study and then provide a brief summary of the anemia-related programs in the region. We then describe the main results of the study, covering the perspectives of adolescents and pregnant adolescents /adolescent mothers, community members and service providers.

### Description of participants

Table 2 provides some basic information on the adolescents and adolescent mothers who participated in the study.

**Table 2:** Characteristics of adolescent participants.

Characteristics	Adolescent (n = 20)	Adolescent mother (n = 18*)
Age range of participants in years Median (IQR)	13–19 15(1.5)	17–19 18(1)
Age range at marriage in years Median (IQR)	N/A	14–19 16(1.75)
Years of education, range Median (IQR)	6–13 9(3)	0–12** 8(3.75)
Years of education husband of adolescent mother Median (IQR)	N/A^+^	0–17^++^ 9(6)

*One data record was incomplete, so we have included 18, rather than 19 participants in this calculation.

**2 data records incomplete for this question.

^+^This information was not collected for the fathers of adolescents.

^++^ Data for 5 cases was incomplete.

Most adolescents’ and adolescent mothers’ families earned a living in unskilled labor or agriculture, or as drivers. Several worked in business or services. According to service providers and community members, poverty is a driver of malnutrition in the area, as well as lack of time due to working conditions, water shortages that undermine hygiene and the common practice of open-air defecation. Illiteracy, lack of nutrition knowledge and illegal child marriage were also seen as relevant contextual factors linked to adolescent malnutrition.

Diets in the area commonly consist of dahl (lentil stew), rotti and rice. Meat and fish were rarely consumed, due to cultural and religious preferences, as well as economic factors. Several adolescents cited preference for a vegetarian diet, although their own families did consume meat. Some adolescents supplement home or school meals with purchased snacks, such as cakes, noodles and other local snack foods.

Most adolescents (of the group who were neither mothers nor pregnant) were attending school at the time of the study. Almost all had career goals, ranging from a vocation as a teacher, to a police officer, to a doctor, although several voiced concerns that they may be expected to marry before finishing school. Many of the adolescent mothers had not completed their education, and were hopeful that their daughters would have better career prospects.

### Description of programs in the research area

Various nutrition programs run by the Ministry of Health and Family Welfare and the Ministry of Women and Child Development were present at community level through the education and primary health care systems, or the *anganwadi centres*. There were minor differences in implementation across the study areas, due in part to modifications as a result of the COVID pandemic. Where possible, the respondents were asked to describe conditions before and after the pandemic.

In government schools, IFA (100mg elemental iron and 500 μg Folic acid) tablets are distributed under the Weekly Iron and Folic Acid Supplementation program (WIFS) for students 11 to 19 years of age. Deworming tablets (Albendazole 400 mg) are given twice a year. All government schools offer a national mid-day meal program, augmented by the school milk program run by the government of Karnataka.

Health and education ministries cooperate to organize nutrition and health counselling and medical check-ups in schools (doctors coupled with community-level health care workers), which may include screening for anemia, with referral to primary health care services if necessary. Specifically for adolescents, SNEHA clinics are run through primary health centers, covering menstrual and reproductive health, nutrition and infectious diseases. Some *anganwadis* also offer food rations for a limited number of adolescent girls not attending school who are living in poverty.

Pregnant women attend antenatal care (ANC) through the primary health center and *anganwadis*, and may also receive home visits from ASHAs. *Anganwadis* provide a hot cooked meal (converted to rations during the pandemic) to pregnant and lactating women, and cash incentives are given to encourage health-seeking behavior. At ANC visits women are screened for anemia and receive IFA tablets (100mg elemental iron and 500μg folic acid daily) and health and nutrition counselling. Further advice is offered through “mothers’ meetings” or community information meetings held at *anganwadis*.

The new Anemia Mukt Bharat program, with specific adolescent-oriented anemia prevention actions, had not yet begun in the area, due to pandemic-related delays.

### Perspectives of adolescents

#### Low understanding or awareness of anemia

We found that almost none of the adolescent girls had specific knowledge about anemia. Several said they had not heard of it. Some did refer to a condition as “less blood” but did not call it anemia. Others described symptoms of anemia, without naming it as such: “When someone does not eat well, they become thin and their blood becomes low. That is malnutrition” (2A5). (We will use “M” to refer to adolescent mothers or pregnant adolescents, and “A” for other adolescents.) Another student affiliated “less blood” with tiredness (2A3). Others mentioned dizziness, or giddiness (3A1). Although many adolescents who were not pregnant were aware of the importance of consuming fruits and vegetables, and many mentioned the nutritional value of home-cooked as compared to street food, very few knew of the specific iron-rich dietary requirements for anemia prevention.

Only one student specifically mentioned “anemia”, explaining that she herself had been hospitalized for “not having enough blood in my body” (3A2). Another student named “hemoglobin”: “I was diagnosed as having less blood… they tested my hemoglobin and said that my hemoglobin is less so they gave some tablet to improve my blood levels” (3A6). Because of their own experiences, these students were more familiar with the terminology. Similarly, these adolescents who had been treated for anemia had stronger understanding of dietary advice. One stated that she had learned to avoid “tea and *kurkure*” (fried snack) and recommended green leafy vegetables, peanuts and jaggery (unrefined sugar), and sprouted pulses (3A2).

#### Low awareness of IFA tablets

Few adolescents were aware that IFA tablets are distributed to prevent anemia. Some adolescents said they had not received IFA tablets at school, and others were unsure, unable to distinguish between IFA tablets and those given for deworming. Few knew what they were for. One adolescent commented, “those are just given to pregnant women” (2A6).

In contrast, others reported that they do receive them, but they are not always consumed, “Some of my friends throw it away, few others take it, some of them take only after the teacher scolds them” (1A1). Adolescents explained that the tablets were taken only because teachers actively enforced it. Some students still managed to avoid it, “we used to put it in the mouth in front of them and later spit it out… It doesn’t have a good taste” (1A7). They had seen students throw them in the bin, or hide them in plants.

However, there were a few who did report taking the tablets. Notably, the student, having been diagnosed with anemia, related the tablets with improved hemoglobin (3A6). One said they were for “health” (1A6), and another said they were to “increase blood in the body” (3A3). Several mentioned that their parents advised them to consume the tablets received at school.

Adolescents named their parents as well as the school as their main sources of health-related information.

### Perspectives of pregnant adolescents and adolescent mothers

#### High awareness of anemia

In comparison, pregnant adolescents and adolescent mothers were more aware of anemia, largely because of their experience with ANC. Testing processes, detection and treatment during ANC visits meant that they gained information about the condition, especially true for those who had been diagnosed with anemia. Some associated it with improved blood: “Your blood has to be good. Your blood reaches the baby also” (2M2).

Pregnant adolescents and young mothers had a higher awareness of iron-rich foods. Several named jaggery, and a few could also list fairly specific foods, all vegetarian: “They [pregnant women] should eat *rotti* (flatbread), rice, *sambar* (lentil vegetable stew) and vegetables like red leafy vegetables, fenugreek, dill… They should eat pulses and millets also like green peas, black-eyed beans and yellow lentils” (3M3).

As well as from family members, pregnant women also mentioned the *anganwadi* and medical centers as places to receive advice. Some even said neighbors had provided information. “Elder people used to tell me to eat green vegetables… They used to tell that eating spinach and beetroot is good for health. ASHA workers also used to tell me to eat green vegetables, millets, fenugreek, spinach…” (1M2). They described how pregnancy brought them into the center of attention:

Everyone advises for our benefit: “By eating these kind of things the blood will increase, weight will increase, the child will be healthy,…if you don’t take, you will create trouble for us” If we don’t take and end up with some complications, they (ASHAs) have to run around for us. They tell it for our benefit so we should take it.” (2M6)

Pregnant adolescents and mothers described how their diets changed during pregnancy, referring not to iron-rich foods, but to vegetables and fruit in general. One said her family took measures to buy more vegetables and give her a bigger share, while another explained that neighbors were requested to share meals with her when there was no time to cook. Several said they stopped consuming junk food due to pregnancy. One described that, in comparison, pre-pregnancy, “We just eat whatever is prepared” (2M1).

#### High awareness of IFA tablets

Most of the pregnant adolescents were aware of IFA tablets, and had received them from health services. Five adolescent mothers did take them, most of whom said they were anemic. Two others had taken them, but discontinued their use. Reasons included dizziness, stomach pains, nausea, and family concerns that the baby would grow too much. Five of the pregnant adolescents/mothers did not take the tablets. One said she “did not need them” (1M2) and another said that they were not supplied (1M5). Most associated strengthened blood with nutritious foods, rather than tablets, and they valued the food ration. As one explained:

I do not think tablets and medicines should be given. I did not take the tablets even though they were given to me by health workers… My family members and others told me not to take those tablets. ASHA workers told me to take those tablets so that I would get strength and the baby will have good growth. But I did not take those tablets thinking that the food I was eating contained everything I needed (3M1).

In summary, pregnancy represents a period when nutrition behaviour changes for adolescents, spurred in part by family and community expectation, but also by medical advice and services which make the condition real, by testing and detecting anemia. Previous to pregnancy, many adolescents did not know about anemia or relate the condition to IFA tablets, and described a situation where students were pressed to take them without being aware of the reason. In a few cases, students were detected with anemia at school, and referred to health services, and gained awareness of the condition in this way.

### Perspectives of community members

#### Some awareness of anemia

Community members spoke more about nutrition and health in general, although some did mention anemia as a specific health problem for female adolescents and linked it to menstruation. Many could name iron-rich foods, such as spinach, figs and peanuts, and recommended sprouted pulses. Most associated adolescent nutrition problems with socioeconomic constraints, such as parents’ long working hours, child marriage, poverty, and lack of education.

#### Some awareness of IFA tablets

Many had heard of IFA tablets, but not all were aware that they were provided in schools. Participants were aware that community opinion was mixed regarding their value, and that some parents were wary of them. “Some mothers ask the children to throw the tablets away” (FDG1CL). Others mentioned that people believe that taking too many “makes the baby grow too big and delivery will not be smooth and easy” (FDG3FM).

#### Views on adolescent dietary habits, before and after pregnancy

Community members described differences in behaviour of school-going adolescents, as compared to those who were pregnant. Before pregnancy, adolescents were seen as unwilling to listen to parents’ advice. Many expressed frustration that adolescents prefer food purchased in the street, rather than traditional home-cooked meals. “They eat them (purchased snacks) outside. Bakery items, food items sold on the carts and road side items they will eat…” They worried that they ate more snacks than meals, even when told not to. Parents felt they did not have control:

My own daughter does not eat *rotti*, nor does she eat raw vegetables. She eats only once in a day. No matter how much we force her to eat, she does not eat at all. She does not have any interest in food. She does not eat at all. Many girls are like this. She is only occupied with her studies and homework and such… (FGD3FM).

Once pregnant, adolescents’ diets were likely to be overseen by family members. Extra fruits and vegetables were purchased for them, and they had priority over others:

Moderator: You all told me that often it is the woman who eats last in the family. If there is a pregnant woman in the family, how does this change?

Participant 5: We have to feed the pregnant woman first. Hot rice, *ghee* (clarified butter).

Moderator: So she is made to eat even before the men?

Participant 4: She is after all two individuals in one at the time. She has a baby in the womb. Hence she is fed first. After she eats, the men eat. (FDG3FM)

Community members stated that socioeconomic conditions meant that the diet was not always ideal, and that at the same time, adolescents were not likely to listen to their parents’ messages were not always inclined to eat what the family offered. These perceptions changed during pregnancy, when families make a concerted effort to prioritize pregnant family members as a necessary measure during pregnancy. Due to cultural expectations around pregnancy, pregnant girls and women were on the receiving end of family-based nutrition and health advice and action. We also observed that pregnancy was perceived within families and communities as requiring health attention, compared to adolescence. The social concern around pregnancy includes diet-related advice and the expectation of increased quantity, quality and diversity of consumption.

### Perspectives of service providers

#### Raising awareness of anemia in adolescents

Before pregnancy, adolescents main encounter with services and information providers on the topic of nutrition was at school, both through teachers and health services staff carrying out school-based medical visits. Although programs were being implemented to varying extents in all schools, service providers were aware that anemia-related advice was not getting through to students. Adolescents were only likely to visit health services for nutrition issues if referred through school-health check-ups. Teachers themselves were involved in nutrition-related activities such as overseeing school meals, and distribution of tablets. Some teachers were aware of anemia. One described the need to take the IFA tablets “for health and strength” (1ST). Another, however, did not know of the word “anemia”, and said none of the students had problems with “reduced blood” (1ST).

Health services providers involved in school health visits described prevention and detection activities. This included checking adolescents’ eyes to detect anemia, and sending them for testing. They spoke of the link between hemoglobin levels and weakness, and named iron-rich foods, such as peanuts, jaggery and sprouts. However, their advice for adolescents was usually a general call to “eat fruit and vegetables” (3ANM). Service providers did not directly promote animal-origin iron-rich foods: due to sensitivity to the cultural preference for vegetarianism and the socioeconomic situation of families. Their advice did not include details on how to ensure sufficient iron consumption in a vegetarian diet, nor the need for supplementation in low-diversity diets. While teachers reported that IFA tablets were indeed distributed, health professionals were concerned that students were not consistently receiving or consuming them.

“When I did school health check-ups, I found out that about 50% of adolescent girls were anemic. Even when they have health problems, they think that they are fine… when they are diagnosed as having anemia they do not agree to take medication” (3MO).

Many described a vigilance-style approach: “If the children are made to swallow, it is fine. But not all schools do that. Some say that they will take the tablets and throw them away” (1MHW). Others reported operational problems, including a lack of continuity of supply of tablets. Adolescent-specific information sessions on nutrition and hygiene, including distribution of sanitary pads, were reported to be offered, but this was not considered to be enough. “We need effective counsellors. And the importance of nutrition and hemoglobin should be stressed in the schools and colleges… Just supplying medicines and noting that they don’t take is of no use” (1AMO). Several suggested that hemoglobin testing, only available in a limited number of schools, should be widespread.

#### Raising awareness of and detecting anemia in pregnant adolescents

Once adolescents became pregnant they were more successfully targeted for nutrition advice and anemia detection through the health sector. They were said to attend ANC visits, where IFA tablets are administered. “Doctors and the medical officer visit the *anganwadi* centers and conduct health check-ups. They identify high risk pregnancies and for such women, the ASHA and AWW conduct home visits” (1CPDO). Community level systems follow-up on pregnant women:

Every month we enquire with the mother and the mother-in-law, whether she has taken tablets or not, whether she has taken food or not…. If the hemoglobin is low, she has to be referred and has to visit the higher center for every check-up as it cannot be handled here. (1ANM).

The main gap was seen to be for adolescents who were not pregnant, as well as adolescents who were in their early phases of pregnancy, as many adolescents arrived at ANC already anemic: “We don’t know their hemoglobin status before pregnancy. They have malnutrition and anemia and it just continues” (1MHW).

#### Raising awareness of anemia in adolescents who do not attend school

Service providers observed that it was especially challenging to reach adolescents who leave school (before or after mandatory schooling up to year ten).

It is very easy to approach those that go to school and colleges because we get them in one place. And when some health department person comes to address them, they will begin to understand… For the ones who don’t go to school, early in the morning, they go the fields for work and their timings are different. Even when we go according to their timings, compared to the positive response we get here, there it is dead opposite. (1AMO)

Despite insistence by education system staff that no adolescents in their jurisdiction had left their mandatory schooling early, many of the adolescent mothers we interviewed had not finished school. Other service providers reported that illegal child marriage did continue in the area despite their efforts, and saw it as highly detrimental to girls’ access to health and education.

In sum, service providers are concerned that just distributing IFA tablets will not lead to results, without improved accompanying information and education. Furthermore, they suggest improved detection systems to reduce the likelihood that anemia is only identified once adolescents become pregnant and attend ANC, where they are subject to detailed follow-up.

## Discussion

We found that actions and information related to anemia were only effectively reaching adolescents once they were pregnant. The importance given to pregnancy as a life phase, both within the family and inside the health system, meant that anemia-related action was most likely during pregnancy. Pregnancy draws attention to the adolescent, both in social circles and in the health system, which facilitates detection of anemia and adherence to dietary advice. Previous to this, awareness of anemia is very low, and anemia detection is irregular. Adolescents do receive nutrition-related information and services at school, but unless they are detected as having anemia, they are not usually aware of the condition, nor necessarily associate it with potential problems during pregnancy. We suggest that measures appropriate for the life-phase of adolescence [22] are required if progress on anemia reduction is to be met.

Table 3 summarizes the findings and indicates that improved school-based interventions and counselling, as well as better outreach to families may help improve their awareness of anemia.

**Table 3:** Findings and recommendations.

	Adolescents		Pregnant adolescents		Recommendations
**Place/ setting**	*Anemia-related attention being received*	*Existing Awareness of anemia and diet*	*Anemia-related attention being received*	*Existing Awareness of anemia and diet*	
**School**	Receive school-based programs, but little information about anemia	**Low awareness** of anemia	NA	NA	Widespread testing for detection and referral, improved counselling about anemia and IFA
**Health services**	Little interaction, unless detected (uncommon)	**Low awareness**, unless detected (uncommon)	detection and treatment	**High awareness** of anemia	Referral after school-based detection
**Family**	Attention from family, but not about anemia	**Low awareness** of dietary needs	Subject to social concern around nutrition	**High awareness** of dietary needs	Improved school outreach to parents to include them in anemia education

Regarding school-based anemia actions, as shown in the first line of Table 3, our findings point to the need to improve school-based awareness. Service providers suggest that attempts to detect anemia are not regular or widespread, and that students are not aware of what they are being asked to consume and why. The lack of knowledge about IFA supplements in the study area echoes that of other studies in India [19,23], which highlight the need for students to receive counselling on the purpose and value of IFA tablets [17]. Evidence shows that adolescent girls’ knowledge of anemia significantly increased after interventions run by dedicated teachers [19]. In our work, schools were named as an important source of information by adolescents, and provided an opportunity to address the communication gap. Research with pregnant woman points to the effectiveness of communication for helping to prevent anemia [24,25]. However, other studies add that knowledge of the condition alone does not drive people to act—they must also perceive themselves at risk [23,26] Research has suggested that using widespread anemia testing would increase awareness [27], an observation that also emerged in our study.

Global literature recognizes the value of distributing supplements in schools [9,28] and the complex issues of adherence [29], and highlights the need for interpersonal communication and support [20,30] and culturally-tailored messaging [20,31]. Research in LMIC settings shows that when supplementation programs include comprehensive communication strategies or targeted information campaigns, they are more able to influence the ingestion of iron supplements in adolescents in LMIC [32,33], and evaluations in India have called for improved community-level outreach and education for behaviour change to help create demand for supplements [34,35].

Within the family and community setting, it was evident that the social importance of pregnancy facilitated attention to diet. Parents of adolescents are knowledgeable about iron-rich traditional foods, but bemoaned adolescents’ lack of interest in nutritious foods and homecooked meals, and felt that they lacked influence over health-related decisions. However, our findings reveal that some adolescents took tablets based on their parents’ advice. Research observes that parents are influential in health-related decision-making [20], and that family food culture is an important influence on adolescent food habits [36,37]. Research in India found that pregnant women were more likely to use IFA tablets and to diversify their diet if family members were part of the strategy [38]. Parental support, involvement and education have been highlighted as ways to raise awareness in adolescents [20,21], as have community-based approaches [39]. Unless communities are concerned about nutrition, it is difficult for health interventions to work [18,26]. Our study also raises questions on how the community can be encouraged to view adolescence as a socially important health period, as is the case with pregnancy, as this would facilitate adolescents’ acceptance of diet-related advice.

In regards to the health system (line 3 on Table 3, above) we described how those who are pregnant are sought out by health services staff. Regular testing and iron supplementation occurs within ANC procedures, including home visits, if necessary. In contrast, previous to pregnancy, adolescent interaction with health sector is infrequent. Service providers note that high numbers of adolescents initiating ANC are anemic. Research in India calls for improved targeting of adolescents and non-pregnant women for nutrition interventions, who despite representing large numbers of cases, are disregarded compared to pregnant women and children [27]. Adolescence is beginning to be prioritized as a separate target group for healthcare interventions, in India [4] and elsewhere [8,9,40] mandating adolescent-specific indicators for nutrition evaluation [5].

In the study area, visits by trained health professionals and local level teams, with referrals to adolescent-specific clinics, were already in place, yet these visits were few and far between. Studies in India have called for better visibility of national programs, improved coordination between government bodies [15] and implementation of a continuous adolescent-oriented service at community level [18]. Increasing school-based anemia education and detection so that adolescents are referred to health services would increase awareness of the condition–as opposed to waiting until pregnancy when most detection occurs.

Anemia advice needs to be tailored to adolescent concerns, interests and needs. At international level, adolescent-specific programming is gaining ground, as are initiatives to raise the profile of adolescence as a critical life-phase: According to the WHO, adolescent health programs should be for the sake of adolescent girls own right to health [22] and should tap values and concerns [41], so as to reach them more effectively. Research on supplementation programs in India suggests that considerations of the specific mental and emotional needs of adolescents, and not only the physical ones, have to be included in health-related programming, and adolescence viewed as a critical stage in the lifecycle [19]. Increased attention to the contextual determinants of adolescent undernutrition is required, beyond the current largely biomedical focus on the issue [42]. Researchers point to the importance of taking into account social norms around adolescence, as well addressing structural issues to create a health-promoting environment, including educational opportunities [38].

Strengths of this study include the combination of both the views of adolescents, and those of the service providers and community members, allowing us to see gaps in attention to adolescents despite the existence of programs. However, our study also faced several limitations. First of all, more male than female researchers were involved. However, this was overcome by ensuring that most adolescent interviews were carried out by female researchers. This study is also limited by our focus on diet-related factors of anemia. We did observe that handwashing is prevalent, but latrine use is low, both of central importance for anemia prevention, but detailed investigation on these topics was beyond the scope of this study.

## Conclusion

Despite the existence of dedicated government programs, we found that awareness about the possibility of adolescent anemia and knowledge about prevention were very limited. Our study points to the lack of prioritization of pre-pregnancy adolescent health: once adolescents became pregnant, they were more successfully targeted for nutrition advice and anemia detection. As well as being in touch with the health system, pregnant adolescents are the focus of care and advice from family, due to social expectations around pregnancy. For adolescents to gain improved awareness of anemia, steps to increase their comprehension of the condition and how it may affect them, including increased detection of anemia, will be required. It is imperative to invest in school-based nutrition education, in cooperation with families, communities and the health sector, so that anemia in adolescence becomes a subject of concern, without waiting for pregnancy. This research contributes to recent positions that argue that adolescence must be considered a specific health category, and that adolescent well-being requires protection, for progress in global health to occur.

## Supporting information

S1 File Inclusivity in global research. (DOCX)
